# Smoking‐Related Mortality in Patients With Early Rheumatoid Arthritis: A Retrospective Cohort Study Using the Clinical Practice Research Datalink

**DOI:** 10.1002/acr.22882

**Published:** 2016-10-06

**Authors:** Rebecca M. Joseph, Mohammad Movahedi, William G. Dixon, Deborah P. M. Symmons

**Affiliations:** ^1^ NIHR Manchester Musculoskeletal Biomedical Research Centre, Central Manchester University Hospital NHS Foundation TrustManchesterUK; ^2^ Arthritis Research UK Centre for Epidemiology, Centre for Musculoskeletal Research, University of ManchesterManchesterUK; ^3^ NIHR Manchester Musculoskeletal Biomedical Research Centre, Central Manchester University Hospital NHS Foundation Trust, and Arthritis Research UK Centre for Epidemiology, Centre for Musculoskeletal Research, University of ManchesterManchesterUK

## Abstract

**Objective:**

To investigate the association between smoking status and smoking cessation with mortality in patients with rheumatoid arthritis (RA).

**Methods:**

An incident cohort of patients with RA was identified using the Clinical Practice Research Datalink, a database of UK primary care electronic medical records. Time‐varying smoking status, years of cessation, and amount smoked were determined from patients' medical records. The date and underlying cause of death were identified by linkage with Office for National Statistics records. The associations between smoking status and smoking cessation with all‐cause and cause‐specific mortality (circulatory disease, all cancers, lung cancer, respiratory disease, and respiratory infection) were investigated using adjusted Cox (all‐cause mortality) and Fine‐Gray (cause‐specific mortality) regression.

**Results:**

The cohort comprised 5,677 patients (median age 61.4 years, 68% women), with 40% as never smokers, 34% former smokers, and 26% current smokers at baseline. Compared to never smoking, current smoking was associated with an increased risk of all‐cause mortality (hazard ratio 1.98 [95% confidence interval (95% CI) 1.56, 2.53]), and mortality due to circulatory disease (subdistribution hazard ratio [SHR] 1.96 [95% CI 1.33, 2.90]) and lung cancer (SHR 23.2 [95% CI 5.15, 105]). Each year of smoking cessation was associated with a decreased risk of all‐cause mortality (former heavy smokers SHR 0.85 [95% CI 0.77, 0.94], former light smokers SHR 0.90 [95% CI 0.84, 0.97]).

**Conclusion:**

Current smoking is associated with an increased risk of all‐cause, cardiovascular, and lung cancer mortality in patients with RA. Each year of cessation is associated with a reduced risk of all‐cause mortality. This information may prove helpful in smoking cessation programs for patients with RA.

## INTRODUCTION

Smoking is a known risk factor for the development of rheumatoid arthritis (RA) 1. The prevalence of smoking is, therefore, higher in patients with RA than in the general population 2. Tobacco smoking is associated with an increased risk of death in the general population, with an estimated 17% of adult deaths in England attributable to smoking in 2013 3. Participants in the Million Women Study who smoked at baseline had approximately 2.7 times increased mortality compared to never smokers 4. Smoking is associated with increased risk of death due to cardiovascular disease (CVD), respiratory infection, and certain forms of cancer, including lung cancer 3, 4. There is also evidence that smoking cessation is associated with a gradual normalization of death rates 4.

Box 1 **Significance & Innovations**

- In this large population‐based study, smoking status was allowed to vary throughout followup, reflecting patients' smoking behavior after a diagnosis of rheumatoid arthritis and allowing duration of smoking cessation to be modelled.
- The risks of all‐cause mortality and death due to lung cancer and circulatory disease were increased in current smokers compared to both never smokers and those who stopped smoking.
- In former smokers the risk of all‐cause mortality decreased for each additional year of smoking cessation, with a greater decrease for patients who had been heavy smokers.
- This information on the risks of continuing to smoke after receiving a diagnosis of rheumatoid arthritis could be useful in smoking cessation programs.

Patients with RA experience an approximately 1.5‐fold increased mortality compared to the general population 5, 6. The most frequent causes of premature mortality in patients with RA are CVD, malignancy, including lung cancer, severe infection, and respiratory diseases 7, 8, 9. Some of the excess mortality in RA is probably attributable to the higher prevalence of smoking. Existing studies have reported an increased risk of mortality associated with smoking (47% per additional pack of cigarettes per day [10] or 83% for ever smoking compared to never smoking [ 7]).

There is some evidence that smoking is associated with an increased risk of death due to cardiopulmonary causes in patients with RA 10. Other studies found that smoking does not predict death from lower respiratory tract infection 11 or cancer 12 in patients with inflammatory arthritis. The question as to whether smoking cessation is beneficial with respect to mortality in patients with RA has not previously been addressed. The aims of this study were to investigate, in patients with RA drawn from a primary care setting, the association between tobacco smoking and all‐cause mortality and mortality due to CVD, all cancers, lung cancer, respiratory infection, and other respiratory disease, and the association between smoking cessation and all‐cause and cause‐specific mortality.

## PATIENTS AND METHODS

#### Setting

The study was set within the Clinical Practice Research Datalink (CPRD), a UK database of anonymized primary care electronic medical records 13. The CPRD has been collecting data since 1987 and currently holds the records of almost 13 million patients, with active patients representing approximately 7% of the UK population. Data held include patient demographics, prescriptions issued, and information about medical conditions, referrals, and clinical tests coded using Read codes 14.

CPRD data can be linked with the Office for National Statistics (ONS) mortality data and the Hospital Episode Statistics (HES) database. To be eligible for linkage, patients are required to have a valid NHS number and be registered with an English general practice participating in the linkage scheme (56% of all CPRD practices). In England, all deaths must be reported to the ONS, and the mortality data available through linkage include the date and underlying cause of death as reported on death certificates (coded using the International Classification of Diseases, Ninth Revision [ICD‐9] and Tenth Revision [ICD‐10] codes). The HES data contain information on hospital inpatient admissions in England and include the reason for admission, coded using ICD‐10 codes, and any procedures carried out, coded using Office of Population Censuses and Surveys Classification of Interventions and Procedures version 4 (OPCS‐4) codes.

#### Population

An incident cohort of adults (ages ≥16) with RA was defined within the CPRD data set. All patients with a Read code for RA in their CPRD records were identified (see Supplementary Table 1, available on the *Arthritis Care & Research* web site at http://onlinelibrary.wiley.com/doi/10.1002/acr.22882/abstract). A validated algorithm shown to have 84% sensitivity and 86% specificity in identifying RA patients (based on fulfilling the 1987 American College of Rheumatology criteria for RA [ 15] or expert opinion [ 16]) was then applied to identify the probable true RA patients (see Supplementary Figure 1, available on the *Arthritis Care & Research* web site at http://onlinelibrary.wiley.com/doi/10.1002/acr.22882/abstract). Linkage with HES and the ONS was requested for the patients identified. Only those patients who were eligible for linkage were included in the final cohort.

To increase the likelihood that only incident RA cases were included, patients were further required to have at least 3 years of up‐to‐standard followup within the CPRD prior to their RA diagnosis. Patients prescribed a disease‐modifying antirheumatic drug (DMARD) more than 3 months before their first recorded RA Read code were also excluded. The date of the first RA Read code or first DMARD prescription (whichever was earliest) was taken to be the date of RA diagnosis.

#### Study window

Data linkage with ONS and HES was available from January 1, 1998 to January 10, 2012, and these dates formed the study window. Patients diagnosed on or after the start of the study window entered the cohort on their RA diagnosis date. Patients were followed until death, leaving their general practice, or the last data collection date for their practice, whichever came first. For those remaining in the cohort, followup was censored on January 10, 2012.

#### Exposure

Smoking status was defined using the CPRD data sets and could vary through time. Followup was divided into periods of never smoking, current smoking, or former smoking. A description of how smoking status was defined is available in Supplementary Figure 2 (available on the *Arthritis Care & Research* web site at http://onlinelibrary.wiley.com/doi/10.1002/acr.22882/abstract).

For former smokers, the duration of smoking cessation (in years) was calculated. The maximum possible duration of known smoking cessation at baseline was 3 years. Any cessation time recorded prior to this limit was not used, as patients had variable amounts of CPRD followup prior to RA diagnosis. The start of cessation was set as the midpoint between the most recent record of an alternative category and the first record of each period of former smoking. To account for a possible interaction with amount smoked, we calculated the amount smoked (heavy versus light) at baseline, using information recorded ≤3 years after baseline. Heavy smoking was defined as smoking more than 20 cigarettes or 10 cigars a day.

#### Outcome

The date and underlying cause of death were identified from the linked ONS data. The following underlying causes of death were studied: cancer (ICD‐9 140‐239, ICD‐10 chapter C), lung cancer (ICD‐9 162, ICD‐10 C34), circulatory disease (ICD‐9 390‐459, ICD‐10 chapter I), lower respiratory tract infection (code list available on ClinicalCodes.org [17]), and other respiratory causes (ICD‐9 460‐519, ICD‐10 chapter J, both minus the infection codes).

#### Covariates

Age, sex, socioeconomic status (SES), year of RA diagnosis, and baseline body mass index (BMI) were identified. SES was defined using the quintile of Townsend score 18, at the ONS small‐area level, based on the patient's postal code. The year of diagnosis, used as a marker for calendar‐year effects, was categorized as before or after January 1, 2000, to coincide with the introduction of biologic therapies for RA in the UK. The data cleaning steps and definition of BMI are described in Supplementary Figure 3 (available on the *Arthritis Care & Research* web site at http://onlinelibrary.wiley.com/doi/10.1002/acr.22882/abstract).

Type 2 diabetes mellitus was identified within the CPRD data sets using a predefined list of Read codes. CVD was identified within the CPRD and HES data sets using Read codes and ICD‐10 codes for myocardial infarction or stroke, and OPCS‐4 codes for CVD‐related procedures such as revascularization. Code lists for diabetes mellitus and CVD are available on ClinicalCodes.org 17. Diabetes mellitus and CVD were included as time‐varying covariates, defined as present from the date of the first ever record onwards, or not present otherwise.

Lists of product codes for DMARDs, oral glucocorticoids, cardiovascular medication (e.g., diuretics, beta‐blockers), aspirin and antiplatelet agents, lipid regulators, and nonsteroidal antiinflammatory drugs were generated based on British National Formulary chapters 19 and clinical knowledge. Use of immunosuppressant DMARDs (methotrexate, azathioprine, cyclosporin, cyclophosphamide, leflunomide, and mycophenolate) was included as a marker of disease severity and thus was defined as present from the date of the first prescription onwards. For oral glucocorticoids, the stop date was calculated using any quantity, daily dose, and duration information provided. For all other included medications, patients were considered as taking the medication for 1 month from the date that each prescription was issued (the standard length for a UK prescription), unless a further prescription had been issued.

#### Statistical analysis

Baseline characteristics were described using the median and interquartile range (IQR) or proportions. Baseline differences between smoking categories were tested using the chi‐square test for categorical variables or the Kruskal‐Wallis test for continuous variables. A significance level of 0.05 was used throughout.

Crude mortality rates were calculated and then adjusted for age and sex by direct standardization of the smoking subgroups to the whole incident cohort. Survival analysis was performed to investigate the association between smoking status and mortality. All‐cause and cause‐specific mortality were examined. Analyses were adjusted first for age and sex, then for all covariates. The association between smoking cessation and mortality was examined in former smokers, including the years of cessation, amount smoked, and their interaction in the models. Years of cessation was entered into the models as a continuous variable, estimating the linear change in risk for each additional year of cessation. The Cox regression model was used to investigate all‐cause mortality. For cause‐specific mortality, where death due to other causes is a competing risk, the Fine‐Gray model was applied 20. The parameters estimated by the Fine‐Gray model (subdistribution hazard ratios [SHR]) reflect the association between a variable and the probability of an outcome but are also influenced by the variable's association with the competing outcomes 21. The proportional hazards assumption was tested for each model by inspecting the Schoenfeld residuals, plotting log (−log[survival]) versus log of survival time plots and looking for interactions between covariates and study time.

For some patients, data were missing for BMI, SES, and amount smoked. Multiple imputation by chained equations 22 was performed using the MI suite in Stata to deal with missing values for BMI and amount smoked. Linear regression was used to impute BMI and logistic regression to impute amount smoked. All predictors included in the final models, including interaction terms, were entered in the imputation model. Twenty data sets were imputed. For the analyses, the results were combined using Rubin's rules 23. All data handling and analysis was performed using Stata/MP software, version 12.1. The protocol for this study was reviewed and approved by the Independent Scientific Advisory Committee (protocol reference 13_159).

## RESULTS

**Figure 1:**
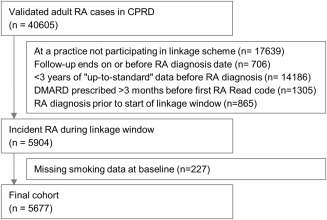
Flowchart showing the definition of the final cohort. RA = rheumatoid arthritis; CPRD = Clinical Practice Research Datalink; DMARD = disease‐modifying antirheumatic drug.

There were 5,904 patients with validated incident RA eligible for inclusion in the cohort (Figure 1). After excluding 227 patients (4%) with missing baseline smoking status, 5,677 patients remained in the cohort, with 3,850 (67.8%) women. The median age at RA diagnosis was 61.4 years (IQR 51.2–71.3) (Table 1). At baseline, 2,288 (40.3%) were never smokers, 1,935 (34.1%) were former smokers, and 1,454 (25.6%) were current smokers. During followup, 917 patients (16%) changed smoking status, including 348 current smokers recorded as stopping smoking after a single attempt. The amount smoked was missing for 27% of former smokers. Of those with this information, 32% ever smoked more than 20 cigarettes a day.

**Table 1:** Baseline characteristics of rheumatoid arthritis inception cohort^a^

Characteristic	All patients	Never smokers	Former smokers	*P*	Current smokers	*P*
No. of patients (%)	5,677	2,288 (40)	1,935 (34)	–	1,454 (26)	–
Women	68	79	60	–	62	–
Pair‐wise comparison		Reference	χ^2^(1df) = 180	0.000	χ^2^(1df) = 124	0.000
Age, median (IQR)	61.4 (51.2–71.3)	60.9 (49.4–72.4)	64.9 (55.8–73.5)	–	58.0 (49.1–66.1)	–
Pair‐wise comparison		Reference	KW(1df) = 51	0.0001	KW(1df) = 42	0.0001
Body mass index, median (IQR)^b^	26.8 (23.8–30.9)	26.6 (23.8–30.7)	27.7 (24.6–31.7)	–	25.8 (22.8–29.5)	–
Pair‐wise comparison		Reference	KW(1df) = 26	0.0001	KW(1df) = 18	0.0001
Townsend score quintile^c^						
1	23.1	28.2	22.1	–	16.4	–
2	24.0	25.4	24.6	–	20.9	–
3	21.8	21.2	21.9	–	22.5	–
4	18.3	15.9	18.9	–	21.4	–
5	12.9	9.4	12.5	–	18.8	–
Pair‐wise comparison		Reference	χ^2^(4df) = 31	0.000	χ^2^(4df) = 137	0.000
Cardiovascular disease	5.3	3.2	8.9	–	4.0	–
Pair‐wise comparison		Reference	χ^2^(1df) = 63	0.000	χ^2^(1df) = 1.9	0.170
Type 2 diabetes mellitus	10.9	9.8	14.8	–	7.3	–
Pair‐wise comparison		Reference	χ^2^(1df) = 26	0.000	χ^2^(1df) = 6.7	0.010
Glucocorticoids	16.7	16.5	18.5	–	14.7	–
Pair‐wise comparison		Reference	χ^2^(1df) = 2.9	0.091	χ^2^(1df) = 2.3	0.126
Cardiovascular medication	26.9	25.7	34.2	–	19.2	–
Pair‐wise comparison		Reference	χ^2^(1df) = 36	0.000	χ^2^(1df) = 21	0.000
Aspirin and antiplatelet agents	8.7	6.9	13.0	–	5.9	–
Pair‐wise comparison		Reference	χ^2^(1df) = 44	0.000	χ^2^(1df) = 1.6	0.200
Lipid regulators	11.8	8.7	18.0	–	8.5	–
Pair‐wise comparison		Reference	χ^2^(1df) = 82	0.000	χ^2^(1df) = 0.01	0.894
NSAIDs	47.1	45.1	44.7	–	53.3	–
Pair‐wise comparison		Reference	χ^2^(1df) = 0.09	0.768	χ^2^(1df) = 24	0.000

a Results are the percentage, unless indicated otherwise. IQR = interquartile range; KW = Kruskal‐Wallis test; NSAIDS = nonsteroidal antiinflammatory drugs.

b Missing in 34.2% of patients.

c Missing in 0.5% of patients.

#### All‐cause mortality

During 26,679 person‐years of followup, 574 patients died, giving a crude mortality rate of 21.5 per 1,000 person‐years (95% confidence interval [95% CI] 19.8, 23.3) (Table 2). After age‐sex standardization, the mortality rates for never, former, and current smokers were 16.2 (95% CI 13.7, 18.6), 22.4 (95% CI 19.7, 25.2), and 31.6 (95% CI 25.3, 37.9) per 1,000 person‐years, respectively, equating to approximately 6 deaths per 1,000 person‐years attributable to former smoking and 15 deaths per 1,000 person‐years attributable to current smoking.

**Table 2:** All‐cause mortality rates stratified by sex and time‐varying smoking status^a^

Smoking status	Periods at risk, no.^b^	Person‐years	Deaths, no.	Crude mortality rate (95% CI)^c^	Adjusted mortality rate (95% CI)^d^	Attributable risk (95% CI)^c^	
Men and women							
All	7,116	26,679	574	21.5 (19.8, 23.3)	22.4 (19.7, 25.2)		–
Never	2,288	10,423	182	17.5 (15.1, 20.2)	16.2 (13.7, 18.6)		Reference
Former	2,898	10,261	261	25.4 (22.5, 28.7)	22.4 (19.7, 25.2)		6.2 (3.9, 8.5)
Current	1,930	5,996	131	21.9 (18.4, 25.9)	31.6 (25.3, 37.9)		15.4 (12.9, 18)
Men							
All	2,379	817	230	28.1 (24.7, 32.0)	26.4 (21.8, 30.9)		–
Never	490	2,012	41	20.4 (15.0, 27.7)	18.5 (12.8, 24.2)		Reference
Former	1,148	3,926	126	32.1 (27.0, 38.2)	26.4 (21.8, 30.9)		7.9 (3.5, 12.3)
Current	741	2,233	63	28.2 (22.0, 36.1)	41.4 (29.6, 53.1)		22.8 (17.7, 27.9)
Women							
All	4,737	18,509	344	18.6 (16.7, 20.7)	20.6 (17.2, 24)		–
Never	1,798	8,411	141	16.8 (14.2, 19.8)	15.1 (12.6, 17.6)		Reference
Former	1,750	6,335	135	21.3 (18.0, 25.2)	20.6 (17.2, 24)		5.5 (2.9, 8.2)
Current	1,189	3,762	68	18.1 (14.3, 22.9)	27.1 (19.7, 34.5)		12.0 (9.1, 14.9)

a 95% CI = 95% confidence interval.

b Patients could change smoking status and therefore may be counted more than once.

c Per 1,000 person‐years.

d Per 1,000 person‐years. Age‐sex adjusted. The male RA mortality rate is adjusted for the age structure of the whole male cohort, and the female mortality rate adjusted for the age structure of the whole female cohort.

After adjustment for age and sex, current smokers had a significantly higher risk of death than never smokers (hazard ratio [HR] 2.18 [95% CI 1.73, 2.76]) or former smokers (HR 1.77 [95% CI 1.43, 2.20]) (Table 3). This risk persisted after full adjustment. Former smokers had a higher mortality risk than never smokers (HR[age.sex adjusted] 1.23 [95% CI 1.01, 1.49]) but this risk became nonsignificant after full adjustment (HR 1.18 [95% CI 0.97, 1.44]).

**Table 3:** Association between time‐varying smoking status and smoking cessation with all‐cause mortality (Cox regression)^a^

	Age‐sex adjusted	Fully adjusted^b^
Time‐varying smoking status		
Current vs. never	2.18 (1.73, 2.76)	1.98 (1.56, 2.53)
Current vs. former	1.77 (1.43, 2.20)	1.68 (1.34, 2.11)
Former vs. never	1.23 (1.01, 1.49)	1.18 (0.97, 1.44)
Smoking cessation		
Per year since cessation, light smoker	0.90 (0.84, 0.95)	0.90 (0.84, 0.97)
Per year since cessation, heavy smoker	0.84 (0.77, 0.92)	0.85 (0.77, 0.94)
Heavy vs. light smoker^c^	2.28 (1.3, 4.0)	1.91 (1.02, 3.57)
Per year since cessation X amount smoked^d^	0.94 (0.84, 1.04)	0.95 (0.84, 1.06)

a Values are the hazard ratio (95% confidence interval).

b Adjusted for age, sex, socioeconomic status, body mass index, cardiovascular disease, type 2 diabetes mellitus, and use of immunosuppressant disease‐modifying antirheumatic drugs, oral glucocorticoids, cardiovascular medications, aspirin/antiplatelet agents, lipid regulators, and nonsteroidal antiinflammatory drugs.

c At the time of cessation.

d The interaction between years since cessation and amount smoked.

In former smokers, the risk of mortality fell significantly with each year since stopping (former heavy smokers HR[full adjusted] 0.85 [95% CI 0.77, 0.94], former light smokers HR[full adjusted] 0.90 [95% CI 0.84, 0.97]). The interaction between cessation years and amount smoked was not significant (HR[full adjusted] 0.95 [95% CI 0.84, 1.06]).

#### Cause‐specific mortality

The major cause of death for the cohort was circulatory disease, with a crude mortality rate of 8.3 per 1,000 person‐years (95% CI 7.3, 9.4) (Table 4). In most cases the age‐sex standardized mortality rates were lowest in never smokers and highest in current smokers. This finding was not true for deaths due to respiratory disease, however, for which former smokers had the highest mortality rate.

**Table 4:** Cause‐specific mortality rates stratified by time‐varying smoking status^a^

Smoking status	Periods at risk, no.^b^	Person‐years	Deaths, no.	Crude mortality rate (95% CI)^c^	Adjusted mortality rate (95% CI)^d^	Attributable risk (95% CI) c
All cancer						
All	7,116	26,679	132	4.9 (4.2, 5.9)	4.9 (4.1, 5.8)	–
Never	2,288	10,423	34	3.3 (2.3, 4.6)	3.3 (2.1, 4.4)	Reference
Former	2,898	10,261	57	5.6 (4.3, 7.2)	4.9 (3.6, 6.2)	1.7 (0.6, 2.7)
Current	1,930	5,996	41	6.8 (5.0, 9.3)	8.3 (5.2, 11.3)	5.0 (3.7, 6.2)
Lung cancer						
All	7,116	26,679	46	1.6 (1.2, 2.2)	1.6 (1.2, 2.1)	–
Never	2,288	10,423	<5	<0.5	0.2 (−0.1, 0.6)	Reference
Former	2,898	10,261	21	1.9 (1.3, 3.0)	1.8 (1.0, 2.6)	1.5 (1.0, 2.0)
Current	1,930	5,996	23	3.7 (2.4, 5.6)	4.4 (2.2, 6.7)	4.2 (3.4, 5.0)
Circulatory disease						
All	7,116	26,679	222	8.3 (7.3, 9.5)	8.3 (7.2, 9.4)	–
Never	2,288	10,423	75	7.2 (5.7, 9.0)	6.5 (4.9, 8.0)	Reference
Former	2,898	10,261	95	9.3 (7.6, 11.3)	8.2 (6.5, 9.9)	1.8 (0.4, 3.2)
Current	1,930	5,996	52	8.7 (6.6, 11.4)	13.3 (9.0, 17.6)	6.8 (5.2, 8.5)
Respiratory disease						
All	7,116	26,679	54	2.0 (1.6, 2.6)	2.0 (1.5, 2.5)	–
Never	2,288	10,423	9	0.9 (0.4, 1.7)	0.9 (0.3, 1.6)	Reference
Former	2,898	10,261	35	3.4 (2.4, 4.8)	3.0 (2.0, 4.0)	2.0 (1.3, 2.8)
Current	1,930	5,996	10	1.7 (0.9, 3.1)	2.6 (0.6, 4.5)	1.7 (1.0, 2.3)
Respiratory infection						
All	7,116	26,679	41	1.5 (1.1, 2.1)	1.5 (1.1, 2.0)	–
Never	2,288	10,423	14	1.3 (0.8, 2.3)	1.2 (0.5, 1.8)	Reference
Former	2,898	10,261	18	1.8 (1.1, 2.8)	1.5 (0.8, 2.3)	0.4 (−0.2, 1.0)
Current	1,930	5,996	9	1.5 (0.8, 2.9)	1.9 (0.6, 3.2)	0.8 (0.1, 1.4)

a 95% CI = 95% confidence interval.

b Patients could change smoking status and therefore may be counted more than once.

c Per 1,000 person‐years.

d Per 1,000 person‐years. Age‐sex adjusted to the structure of the whole cohort.

This pattern was reflected in the results of the Fine‐Gray regression models (Table 5), where the risks of mortality due to circulatory disease and lung cancer were increased in current compared to both never and former smokers. The results were not significant for all‐site cancer or respiratory infection. The risk of mortality due to lung cancer was increased in former compared to never smokers (SHR[full adjusted] 7.78 [95% CI 1.73, 35.0]); this finding was also the case for mortality due to respiratory disease, although the result was not statistically significant.

**Table 5:** Association between time‐varying smoking status and smoking cessation with cause‐specific mortality (Fine‐Gray regression)^a^

Cause of death	Age‐sex adjusted	Fully adjusted^b^
All cancer		
Smoking status		
Current vs. never	1.34 (0.53, 3.20)	1.56 (0.83, 2.94)
Current vs. former	2.04 (0.78, 5.31)	1.48 (0.93, 2.35)
Former vs. never	0.64 (0.27, 1.50)	1.06 (0.63, 1.77)
Smoking cessation		
Per year since cessation, light smoker	0.95 (0.82, 1.10)	0.99 (0.84, 1.18)
Per year since cessation, heavy smoker	0.79 (0.64, 0.97)	0.81 (0.63, 1.05)
Heavy vs. light smoker^c^	4.15 (1.07, 16.1)	4.43 (0.71, 27.8)
Per year since cessation of X amount smoked^d^	0.83 (0.63, 1.09)	0.82 (0.59, 1.14)
Lung cancer		
Smoking status		
Current vs. never	24.8 (5.72, 108)	23.2 (5.15, 105)
Current vs. former	3.21 (1.66, 6.20)	2.98 (1.47, 6.06)
Former vs. never	7.73 (1.76, 34.0)	7.78 (1.73, 35.0)
Smoking cessation		
Per year since cessation, light smoker	1.04 (0.79, 1.36)	1.06 (0.76, 1.48)
Per year since cessation, heavy smoker	0.84 (0.53, 1.34)	0.91 (0.61, 1.37)
Heavy vs. light smoker^c^	3.96 (0.34, 45.5)	2.77 (0.25, 30.5)
Per year since cessation of X amount smoked^d^	0.81 (0.45, 1.46)	0.86 (0.51, 1.44)
Circulatory disease		
Smoking status		
Current vs. never	2.13 (1.47, 3.08)	1.96 (1.33, 2.90)
Current vs. former	1.88 (1.32, 2.68)	1.88 (1.30, 2.72)
Former vs. never	1.13 (0.83, 1.54)	1.05 (0.76, 1.44)
Smoking cessation		
Per year since cessation, light smoker	0.99 (0.87, 1.13)	0.98 (0.82, 1.18)
Per year since cessation, heavy smoker	0.80 (0.70, 0.91)	0.76 (0.65, 0.89)
Heavy vs. light smoker^c^	1.46 (0.33, 6.40)	1.56 (0.32, 7.43)
Per year since cessation of X amount smoked^d^	0.81 (0.67, 0.98)	0.77 (0.61, 0.98)
Respiratory disease		
Smoking status		
Current vs. never	0.64 (0.74, 5.57)	0.54 (0.06, 4.62)
Current vs. former	0.32 (0.04, 2.52)	0.26 (0.03, 1.98)
Former vs. never	2.01 (0.60, 6.81)	2.11 (0.60, 7.40)
Smoking cessation		
Per year since cessation, light smoker	0.99 (0.83, 1.17)	1.00 (0.85, 1.18)
Per year since cessation, heavy smoker	0.78 (0.61, 1.00)	0.79 (0.62, 1.00)
Heavy vs. light smoker^c^	5.07 (1.21, 21.3)	5.47 (1.16, 25.8)
Per year since cessation of X amount smoked^d^	0.79 (0.58, 1.07)	0.79 (0.59, 1.06)
Respiratory infection		
Smoking status		
Current vs. never	2.32 (1.00, 5.35)	2.13 (0.84, 5.37)
Current vs. former	2.06 (0.92, 4.61)	2.02 (0.83, 4.93)
Former vs. never	1.13 (0.56, 2.28)	1.05 (0.52, 2.14)
Smoking cessation		
Per year since cessation, light smoker	0.80 (0.55, 1.17)	0.60 (0.37, 0.95)
Per year since cessation, heavy smoker	1.07 (0.88, 1.31)	1.02 (0.85, 1.22)
Heavy vs. light smoker^c^	3.17 (0.30, 33.3)	1.14 (0.22, 5.99)
Per year since cessation of X amount smoked^d^	1.33 (0.84, 2.12)	1.72 (0.98, 3.01)

a Values are the subdistribution hazard ratio (95% confidence interval).

b Adjusted for age, sex, socioeconomic status, body mass index, cardiovascular disease, type 2 diabetes mellitus, and use of immunosuppressant disease‐modifying antirheumatic drugs, oral glucocorticoids, cardiovascular medications, aspirin/antiplatelet agents, lipid regulators, and nonsteroidal antiinflammatory drugs.

c At the time of cessation.

d The interaction between years since cessation and amount smoked.

In former heavy smokers, each year of smoking cessation was associated with a decrease in the risk of mortality due to cancer, circulatory disease, and respiratory disease, although this finding was significant only for deaths due to circulatory disease (Table 5). Each year of smoking cessation was associated with a decrease in the risk of death due to respiratory infection for former light smokers.

## DISCUSSION

In this incident cohort of 5,677 patients with RA, tobacco smoking was associated with an increased risk of mortality. Current smokers had more than double the risk of dying from any cause during followup compared to those who had never smoked. Current smokers also had significantly increased risk of dying due to circulatory disease or lung cancer.

Strengths of the study include that it is a large population‐based inception cohort of patients with RA, with sufficient power to examine both all‐cause and cause‐specific mortality and that, via record linkage, mortality data for the cohort are complete. The CPRD patient population is broadly representative of the UK population 14, 24. While our cohort was limited to practices with linkage to the English HES and ONS databases, leading to 43% of patients being excluded, the cohort should be representative of all English patients with RA. A further strength is that smoking status is treated in a time‐varying fashion, as smoking status changed for 16% of patients during followup. Previous studies of smoking and mortality in RA have only used smoking status at baseline. By allowing smoking status to vary throughout followup, our study reflects more accurately the patients' exposure. This is also the first study to use a competing risks model to examine the relationship between smoking and cause‐specific mortality in RA.

An increased risk of all‐cause mortality associated with smoking in patients with RA has been demonstrated in some 7, 10, but not all 8, 25, previous studies. One of the 2 studies which reported no association, the Early Rheumatoid Arthritis Study 25, collected smoking data retrospectively, and data were missing for 37% of patients. The proportion with missing smoking was probably higher in those who had died and so those who were analyzed may have been healthier or at reduced risk of smoking‐related mortality. Patients in the second study, the Norfolk Arthritis Register cohort 8, were younger at diagnosis than our CPRD cohort (median age at onset 53 versus 61 years) and included all patients with early inflammatory arthritis; the proportion of smoking‐related deaths may have been lower in such a cohort.

Both previous studies that examined the association between smoking and CVD mortality failed to find a significant association 25, 26. In the study from the Mayo clinic 26, current smokers had double the risk of cardiovascular death compared to never smokers, although this finding did not reach statistical significance (HR 1.98 [95% CI 0.93, 4.24]), perhaps because the study was underpowered (603 patients). We did not find a significant association between current smoking and deaths due to all‐site cancer but found a strong association between current smoking and deaths due to lung cancer. Mattey et al 27, also found no association between smoking and mortality due to all cancers but reported a strong association between smoking and deaths due to lung cancer in patients with RA (HR 8.97 [95% CI 1.71, 37.0]).

We found an increased risk of death from respiratory infection among current smokers, although this did not reach statistical significance as the number of deaths (n = 41) was low. For other respiratory mortality, former smokers had a nonsignificant increased risk of death compared to both current and never smokers. This risk could be because patients with pre‐existing respiratory disorders are more likely to stop smoking. Former smokers were also more likely to have had a CVD event or to be treated with cardiovascular medications at baseline, which suggests that the risk or presence of smoking‐related outcomes could be motivating patients to stop smoking. Therefore, those who continue to smoke may be comparatively healthy.

A decrease in risk of all‐cause mortality and death due to circulatory disease was seen for each year of smoking cessation, particularly for those who had been heavy smokers. These results are in agreement with most of the literature from the general population 4, 28. The SHRs for all‐cancer and respiratory deaths were similar to those for circulatory disease but failed to reach statistical significance, while the risk of death due to respiratory infection was found to decrease for each year of cessation in former light smokers only. There is some evidence for a benefit of smoking cessation for specific causes of death in other populations. In the Lung Health study, which comprised individuals who were, on average, heavy smokers with evidence of airway obstruction, mortality rates fell progressively from those who continued to smoke, to intermittent quitters and to those who successfully stopped smoking 29. This pattern was seen for deaths due to coronary heart disease, CVD, and lung cancer (but not other forms of cancer).

Limitations of our study include the limited followup time, in addition to which, reducing the sample size and linkage with HES and the ONS reduced the length of the study window. Mean followup was 4.7 years, with a range of 0–14 years. The association between smoking and mortality may alter as the disease progresses. Another limitation is reliance on an algorithm to identify cases of RA 16 rather than being able to apply classification criteria on an individual basis. This is a general limitation for research set within routinely collected health data, in which it is usually not feasible or possible to verify individual diagnoses. The algorithm used has a sensitivity and specificity of over 80%, and we consider the patients included in the cohort to be highly probable cases, while acknowledging that some patients will have been wrongly excluded or included. In addition, this inception cohort is possibly contaminated by prevalent cases. Patients with prevalent RA may have been included as incident cases if they had no significant medical events or changes in medication in the 3 years prior to inclusion. Our definition of RA diagnosis date introduces some immortal time, up to a maximum of 3 months per patient. Those who enter upon DMARD prescription survive at least until receiving an RA Read code. As this result is unlikely to be associated with smoking behavior, we believe it is unlikely to be a source of bias.

Furthermore, misclassification in smoking exposure could result from using general practitioner (GP) records. Smoking information in the CPRD will be affected by how frequently patients visit their GP, how often smoking information was recorded at consultations, how accurately patients report their smoking behavior, and how accurately practice staff record the information. There is some evidence that current smoking is more completely and accurately captured by UK GPs than never and former smoking 30, 31. Former smoking in particular was poorly captured in the 1990s, but there is evidence of an improvement over time 31, 32. Patients may underreport their smoking habits to their health care providers, and some cessation attempts could be missed. Overall, the effect of misclassification is likely to make the exposure groups more similar to each other, which would lead to underestimation of any associations.

In conclusion, patients with RA who smoked tobacco were at significantly increased risk of death from all‐causes and death due to circulatory disease or lung cancer compared to RA patients who never smoked and those who stopped smoking. The risk of all‐cause mortality fell significantly for each year of smoking cessation. This information may be of value to those developing smoking cessation programs for patients with RA 33.

## AUTHOR CONTRIBUTIONS

All authors were involved in drafting the article or revising it critically for important intellectual content, and all authors approved the final version to be submitted for publication. Dr. Symmons had full access to all of the data in the study and takes responsibility for the integrity of the data and the accuracy of the data analysis.

**Study conception and design**. Joseph, Movahedi, Symmons.

**Acquisition of data**. Movahedi.

**Analysis and interpretation of data**. Joseph, Movahedi, Dixon, Symmons.

## Supporting information

Supplementary Figure 1

Supplementary Figure 2

Supplementary Figure 3

Supplementary Table 1
