# Supplementary material for: Smoking‐Related Mortality in Patients With Early Rheumatoid Arthritis: A Retrospective Cohort Study Using the Clinical Practice Research Datalink
Source: Arthritis Care Res (Hoboken). 2016 Oct 6;68(11):1598–606. doi: 10.1002/acr.22882 (PMC5091627; doi:10.1002/acr.22882)
Supplement: Supplementary file 1 — Supplementary Figure 1 [file ACR-68-1598-s001.docx]

**Supplementary Figure 1.** Validation steps to identify patients with rheumatoid arthritis. RA, rheumatoid arthritis; CPRD, Clinical Practice Research Datalink; n, number; DMARD, disease-modifying antirheumatic drug. Method adapted from Thomas et al. *Arthritis & Rheumatism* 2008, 59:1314-21 by Dr Mohammad Movahedi, ARUK Centre for Epidemiology, the University of Manchester. RA Read codes are given in Supplementary Table 1.

**All patients with a Read code for RA in CPRD**

n=45206

**1**

A and B and C

n=32416

**2**

DMARD prescription following an RA Read code, with no alternative indication (cancer, inflammatory bowel disease or psoriasis) recorded in the 5 years prior to the first DMARD prescription

n=33442

**RA according to algorithm**

1 OR 2

**n=44430**

**A**

Strong RA Read code (seropositive RA, erosive RA or "rheumatoid arthritis", rather than systemic manifestations of RA or seronegative RA)

n=43640

**B**

Two or more RA Read codes on different days

n=33510

**C**

No Read code for an alternative diagnosis (e.g. gout, ankylosing spondylitis, reactive arthritis) after the last RA Read code

n=44023
