# Supplementary material for: Smoking‐Related Mortality in Patients With Early Rheumatoid Arthritis: A Retrospective Cohort Study Using the Clinical Practice Research Datalink
Source: Arthritis Care Res (Hoboken). 2016 Oct 6;68(11):1598–606. doi: 10.1002/acr.22882 (PMC5091627; doi:10.1002/acr.22882)
Supplement: Supplementary file 2 — Supplementary Figure 2 [file ACR-68-1598-s002.docx]

1. Identify smoking information from within the CPRD. Three sources: Read codes (status / amount / advice / referrals etc.), additional clinical information (status / amount / advice) and prescriptions for smoking cessation therapy.
2. Combine information from three sources into records: there may be multiple records per day.
3. Set a status for each record based on the combined information, resolving conflicts based on defined rules (e.g. Not Current but prior therapy, therefore Former).
4. Collapse to give a status for each day, resolving conflicts based on defined rules.
5. Clean longitudinal data: Not Current to Never or Former; Ever to Current (or dropped); defined rules for impossible Never.
6. Collapse to a start/stop matrix. Start date = first instance of a new status, stop date = start of next status or follow-up end.

**Supplementary Figure 2.** Summary of steps taken to define smoking status using data from the Clinical Practice Research Datalink.
