# Supplementary material for: Smoking‐Related Mortality in Patients With Early Rheumatoid Arthritis: A Retrospective Cohort Study Using the Clinical Practice Research Datalink
Source: Arthritis Care Res (Hoboken). 2016 Oct 6;68(11):1598–606. doi: 10.1002/acr.22882 (PMC5091627; doi:10.1002/acr.22882)
Supplement: Supplementary file 3 — Supplementary Figure 3 [file ACR-68-1598-s003.docx]

1. Clean height: set to missing if <1.21m, >2.14m or recorded prior to age 18
2. Calculate median height for each patient
3. Clean weight: set to missing if <20kg or >450kg
4. Clean weight: identify further outliers by fitting a random intercepts model (adjusting for age and gender) regressing weight on time (grouping: patient) and calculating standardised residuals. Set to missing
5. Drop weights recorded more than 2 years prior to or 1 year after RA diagnosis date.
6. Keep only the weight recorded closest to the RA diagnosis date
7. Calculate BMI (weight / (height*height))

**Supplementary Figure 3.** Defining BMI within the Clinical Practice Research Datalink. RA rheumatoid arthritis, BMI body mass index. Method developed by Ruth Costello, ARUK Centre for Epidemiology, The University of Manchester.
