# Supplementary material for: Smoking‐Related Mortality in Patients With Early Rheumatoid Arthritis: A Retrospective Cohort Study Using the Clinical Practice Research Datalink
Source: Arthritis Care Res (Hoboken). 2016 Oct 6;68(11):1598–606. doi: 10.1002/acr.22882 (PMC5091627; doi:10.1002/acr.22882)
Supplement: Supplementary file 4 — Supplementary Table 1 [file ACR-68-1598-s004.docx]

**Supplementary Table 1.** Rheumatoid arthritis Read codes, indicating "strong" codes

| **Read code** | **Description** | **Strong?** |
| --- | --- | --- |
| 14G1.00 | H/O: rheumatoid arthritis | strong |
| 38DZ.00 | Disease activity score in rheumatoid arthritis | strong |
| 66H..13 | Rheumatoid arthrit. monitoring | strong |
| N005.00 | Adult Still's Disease | strong |
| N040.00 | Rheumatoid arthritis | strong |
| N040000 | Rheumatoid arthritis of cervical spine | strong |
| N040100 | Other rheumatoid arthritis of spine | strong |
| N040200 | Rheumatoid arthritis of shoulder | strong |
| N040400 | Rheumatoid arthritis of acromioclavicular joint | strong |
| N040500 | Rheumatoid arthritis of elbow | strong |
| N040600 | Rheumatoid arthritis of distal radio-ulnar joint | strong |
| N040700 | Rheumatoid arthritis of wrist | strong |
| N040800 | Rheumatoid arthritis of MCP joint | strong |
| N040900 | Rheumatoid arthritis of PIP joint of finger | strong |
| N040A00 | Rheumatoid arthritis of DIP joint of finger | strong |
| N040B00 | Rheumatoid arthritis of hip | strong |
| N040C00 | Rheumatoid arthritis of sacro-iliac joint | strong |
| N040D00 | Rheumatoid arthritis of knee | strong |
| N040F00 | Rheumatoid arthritis of ankle | strong |
| N040G00 | Rheumatoid arthritis of subtalar joint | strong |
| N040H00 | Rheumatoid arthritis of talonavicular joint | strong |
| N040J00 | Rheumatoid arthritis of other tarsal joint | strong |
| N040K00 | Rheumatoid arthritis of 1st MTP joint | strong |
| N040L00 | Rheumatoid arthritis of lesser MTP joint | strong |
| N040S00 | Rheumatoid arthritis - multiple joint | strong |
| N040T00 | Flare of rheumatoid arthritis | strong |
| N047.00 | Seropositive errosive rheumatoid arthritis | strong |
| N04X.00 | Seropositive rheumatoid arthritis, unspecified | strong |
| N04y200 | Adult-onset Still's disease | strong |
| Nyu1100 | [X]Other seropositive rheumatoid arthritis | strong |
| Nyu1200 | [X]Other specified rheumatoid arthritis | strong |
| Nyu1G00 | [X]Seropositive rheumatoid arthritis, unspecified | strong |
| 7P20300 | Delivery of rehabilitation for rheumatoid arthritis |  |
| F371200 | Polyneuropathy in rheumatoid arthritis |  |
| F396400 | Myopathy due to rheumatoid arthritis |  |
| G5y8.00 | Rheumatoid myocarditis |  |
| G5yA.00 | Rheumatoid carditis |  |
| H570.00 | Rheumatoid lung |  |
| N04..00 | Rheumatoid arthritis and other inflammatory polyarthropathy |  |
| N040N00 | Rheumatoid vasculitis |  |
| N040P00 | Seronegative rheumatoid arthritis |  |
| N040R00 | Rheumatoid nodule |  |
| N041.00 | Felty's syndrome |  |
| N042.00 | Other rheumatoid arthropathy + visceral/systemic involvement |  |
| N042100 | Rheumatoid lung disease |  |
| N042200 | Rheumatoid nodule |  |
| N042z00 | Rheumatoid arthropathy + visceral/systemic involvement NOS |  |
| N04y000 | Rheumatoid lung |  |
| N04y011 | Caplan's syndrome |  |
| N04y012 | Fibrosing alveolitis associated with rheumatoid arthritis |  |
